# Complete genome sequence of *Thermocrinis albus* type strain (HI 11/12^T^)

**DOI:** 10.4056/sigs.761490

**Published:** 2010-03-30

**Authors:** Reinhard Wirth, Johannes Sikorski, Evelyne Brambilla, Monica Misra, Alla Lapidus, Alex Copeland, Matt Nolan, Susan Lucas, Feng Chen, Hope Tice, Jan-Fang Cheng, Cliff Han, John C. Detter, Roxane Tapia, David Bruce, Lynne Goodwin, Sam Pitluck, Amrita Pati, Iain Anderson, Natalia Ivanova, Konstantinos Mavromatis, Natalia Mikhailova, Amy Chen, Krishna Palaniappan, Yvonne Bilek, Thomas Hader, Miriam Land, Loren Hauser, Yun-Juan Chang, Cynthia D. Jeffries, Brian J. Tindall, Manfred Rohde, Markus Göker, James Bristow, Jonathan A. Eisen, Victor Markowitz, Philip Hugenholtz, Nikos C. Kyrpides, Hans-Peter Klenk

**Affiliations:** 1 University of Regensburg, Archaeenzentrum, Regensburg, Germany; 2 DSMZ – German Collection of Microorganisms and Cell Cultures GmbH, Braunschweig, Germany; 3 DOE Joint Genome Institute, Walnut Creek, California, USA; 4 Los Alamos National Laboratory, Bioscience Division, Los Alamos, New Mexico, USA; 5 Biological Data Management and Technology Center, Lawrence Berkeley National Laboratory, Berkeley, California, USA; 6 Oak Ridge National Laboratory, Oak Ridge, Tennessee, USA; 7 HZI – Helmholtz Centre for Infection Research, Braunschweig, Germany; 8 University of California Davis Genome Center, Davis, California, USA

**Keywords:** microaerophilic, (hyper-)thermophile, chemolithoautotrophic, biogeochemistry, non-sporeforming, Gram-negative, flagellated, non-pathogen, *Aquificaceae*, GEBA

## Abstract

*Thermocrinis albus* Eder and Huber 2002 is one of three species in the genus *Thermocrinis* in the family *Aquificaceae*. Members of this family have become of significant interest because of their involvement in global biogeochemical cycles in high-temperature ecosystems. This interest had already spurred several genome sequencing projects for members of the family. We here report the first completed genome sequence a member of the genus *Thermocrinis* and the first type strain genome from a member of the family *Aquificaceae*. The 1,500,577 bp long genome with its 1,603 protein-coding and 47 RNA genes is part of the *** G****enomic* *** E****ncyclopedia of* *** B****acteria and* *** A****rchaea * project.

## Introduction

Strain HI 11/12^T^ (= DSM 14484 = JCM 11386) is the type strain of the species *Thermocrinis albus* [1]. Officially, the genus *Thermocrinis* currently contains three species [2], however, it should be noted that at the time of writing, the 16S rDNA sequence of the type strain of *Thermocrinis ruber* held in the DSMZ open collection as DSM 12173 does not correspond with that published under AJ005640. The generic name derives from the Greek word ‘therme’, meaning ‘heat’, and the Latin word ‘crinis’, hair, meaning ‘hot hair’, referring to the long hair-like filamentous cell structures found in the high-temperature environments, such as hot-spring outlets [3]. These long filaments are formed under conditions where there is a continuous flow of medium. The species name is derived from the Greek word ‘alphos’, white, referring to the cell color [1]. Strain HI 11/12^T^ has been isolated from whitish streamers in Hveragerthi, Iceland [3]. Other strains of the species have been isolated from further high-temperature habitats in Iceland, but also in Kamchatka, Russia [1]. Members of the genus *Thermocrinis* appear to play a major ecological role in global biochemical cycles in such high-temperature habitats [4-7]. As currently defined the genus does not appear to form a monophyletic group, suggesting that further taxonomic work is necessary.

The large interest in the involvement of members of the family *Aquificaceae* in global biogeochemical cycles in high-temperature ecosystems made them attractive targets for early genome sequencing, *e.g.* ‘*Aquifex aeolicus*’ [8], the third hyperthermophile whose genome was already decoded in 1998 [9]. Like ‘*A. aeolicus*’ (a name that was never validly published) strain VF5 [10], *Hydrogenobaculum* sp. Y04AAS1 (CP001130, JGI unpublished) and *Hydrogenivirga* sp. 128-5-R1-1 (draft, Moore Foundation) do not represent type strains. Here we present a summary classification and a set of features for *T. albus* HI 11/12^T^, together with the description of the complete genomic sequencing and annotation.

## Classification and features

Only four cultivated strains are reported for the species *T. albus* in addition to HI 11/12^T^: Strains H7L1B and G3L1B from the same team that isolated HI 11/12^T^ [1], and SRI-48 (AF255599) from hot spring microbial mats [11]. All three strains originate from Iceland and share 98.9-99.7% 16S rRNA sequence identity with HI 11/12^T^. The only non-Icelandic isolate, UZ23L3A (99.2%), originates from Kamchatka (Russia) [1]. Almost all uncultured clones also originate from Iceland: clones KF6 and HV-7 (GU233821 and GU233840, >99%) from water-saturated sediment in the Krafla and Hveragerdi geothermal systems, respectively. Clones GY1-1 and GY1-2 (GU233809, GU233812, >99%) from water-saturated sediment Geysir hot springs; clone SUBT-1 (AF361217, 99.2%) from subterranean hot springs [12], and clone PIce1 (AF301907, 99.3%) as the dominant clone from a blue filament community of a thermal spring. Only clone PNG_TB_4A2.5H2_B11 (EF100635, 95.9%) originated from a non-Icelandic source: a heated, arsenic-rich sediment of a shallow submarine hydrothermal system on Ambitle Island, Papua New Guinea. According to the original publication the 16S rRNA of the type strain of the closest related species within the genus, *T. ruber* [3], shares 95.2% sequence identity, whereas the type strains from the closest related genus, *Hydrogenobacter*, share 94.7-95.0% sequence identity, as determined with EzTaxon [13]. However, as noted above the 16S rDNA sequence of the *T. ruber* strain held in the DSMZ (DSM 12173) does not correspond with the sequence deposited (AJ005640). Environmental samples and metagenomic surveys featured in the NCBI database contain not a single sequence with >88% sequence identity (as of February 2010), indicating that the species *T. albus* might play a rather limited and regional role in the environment.

**Figure 1:**
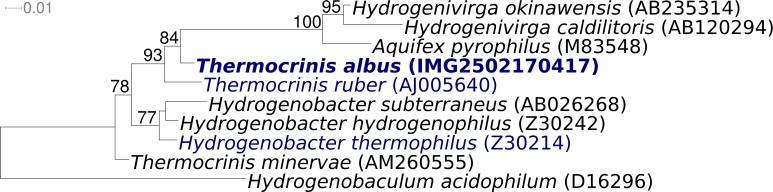
Phylogenetic tree highlighting the position of *T. albus* HI 11/12^T^ relative to the other type strains within the family *Aquificaceae*. The tree was inferred from 1,439 aligned characters [14,15] of the 16S rRNA gene sequence under the maximum likelihood criterion [16] and rooted in accordance with the current taxonomy. The branches are scaled in terms of the expected number of substitutions per site. Numbers above branches are support values from 250 bootstrap replicates [17] if larger than 60%. Lineages with type strain genome sequencing projects registered in GOLD [18] are shown in blue, published genomes in bold. Note that the sequence AJ005640 does not correspond with that from the type strain of *T. ruber* deposited as DSM 12173.

Figure 1 shows the phylogenetic neighborhood of *T. albus* HI 11/12^T^ in a 16S rRNA based tree. The sequence of the single 16S rRNA gene copy in the genome differs by seven nucleotides from the previously published 16S rRNA sequence generated from DSM 14484 (AJ278895), which contains two ambiguous base calls.

**Figure 2:**
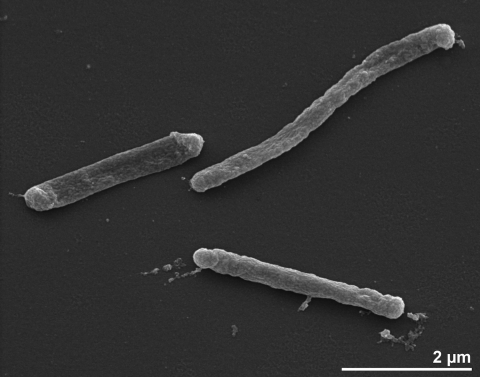
Scanning electron micrograph of *T. albus* HI 11/12^T^

When grown in the laboratory in a continuous flow of medium, for example in a glass chamber [1], strain HI 11/12^T^ exhibits filamentous growth with a length of 10-60 µm [1]. When grown in static culture, the strain grows singly or in pairs [1]. The cells are short rods with 0.5-0.6 µm in width by 1-3 µm in length and motile by means of a monopolar monotrichous flagellum [1] (Figure 2 and Table 1). However, no flagella are visible in Figure 2. A regularly arrayed surface layer protein was not observed [1].

**Table 1:** Classification and general features of *T. albus* HI 11/12^T^ according to the MIGS recommendations [19]

**MIGS ID**	**Property**	**Term**	**Evidence code**
	Current classification	Domain *Bacteria*	TAS [20]
		Phylum *Aquificae*	TAS [21]
		Class *Aquificae*	TAS [21]
		Order *Aquificales*	TAS [22]
		Family *Aquificaceae*	TAS [23]
		Genus *Thermocrinis*	TAS [3]
		Species *Thermocrinis albus*	TAS [1]
		Type strain HI 11/12	TAS [1]
	Gram stain	Gram negative	TAS [1]
	Cell shape	both filament and rod	TAS [1]
	Motility	monopolar monotrichous flagellation	TAS [1]
	Sporulation	non-sporulating	TAS [1]
	Temperature range	55–89°C	TAS [1]
	Optimum temperature	not determined	TAS [1]
	Salinity	≤ 0.7%	TAS [1]
MIGS-22	Oxygen requirement	aerobic	TAS [1]
	Carbon source	CO_2_, no organic carbon source reported	TAS [1,24]
	Energy source	chemolithoautotrophic	TAS [1]
MIGS-6	Habitat	hot spring	TAS [1]
MIGS-15	Biotic relationship	free living	TAS [1]
MIGS-14	Pathogenicity	not reported	TAS [3,25]
	Biosafety level	1	TAS [25]
	Isolation	hot streamlet	TAS [1]
MIGS-4	Geographic location	Hverageroi, Iceland	TAS [1]
MIGS-5	Sample collection time	1998 or before	TAS [3]
MIGS-4.1 MIGS-4.2	Latitude Longitude	64, -21.2	NAS
MIGS-4.3	Depth	unknown	
MIGS-4.4	Altitude	30 m	NAS

Evidence codes - IDA: Inferred from Direct Assay (first time in publication); TAS: Traceable Author Statement (i.e., a direct report exists in the literature); NAS: Non-traceable Author Statement (i.e., not directly observed for the living, isolated sample, but based on a generally accepted property for the species, or anecdotal evidence). These evidence codes are from of the Gene Ontology project [26]. If the evidence code is IDA, then the property was directly observed for a live isolate by one of the authors or an expert mentioned in the acknowledgements.

Strain 11/12^T^ is microaerophilic with oxygen as electron acceptor [1]. Strain 11/12^T^ appears to be strictly chemolithoautotrophic [1]. This differentiates *T. albus* from its two sister species *T. ruber* and *T. minervae*, which both can also grow chemoorganoheterotrophically [3,24]. Strain 11/12^T^ grows optimally under microaerophilic conditions when hydrogen and sulfur are present simultaneously as electron donors [1], however, no growth is observed on nitrate. Physiological characteristics such as the wide temperature preference are reported in Table 1.

### Chemotaxonomy

The cell wall of strain 11/12^T^ contains *meso*-diaminopimelic acid [1]. There are no reports on the presence of a lipopolysaccharide in the typical Gram-negative cell wall, although there are reports of an LPS in *Aquifex pyrophilus* [27,28]. Cellular polyamines are important to stabilize cellular nucleic acid structure as a major function, and may function in thermophilic eubacteria as important chemotaxonomic markers [29]. In the genus *Thermocrinis*, the major polyamines are spermidine and a quaternary branched penta-amine, N^4^-bis(aminopropyl)-norspermidine [29].

The major fatty acids in strain HI 11/12^T^ are *cyclo*-C_21:0_ (42%, 2 isomers), C_18:0_ (14%), C_20:1 cΔ11_ (10.7%), C_20:1 cΔ13_ (8.2%), C_20:1 tΔ11_ (5.4%), and C_20:1 tΔ13_ (3.5%) [30]. All other fatty acids are below 2.9%) [30]. The polar lipids are based on ester linked fatty acids (diacyl glycerols) and monoether (probably in the form of monoester-monoether glycerols), in which the ether linked side chain includes C_18:0_ (78.5%), C_20:0_ (2.0%), C_20:1_ (17.6%) and C_21:0_ (1.9%) side chains [30].

*T. albus* belongs to a group of organisms where characteristic sulfur containing napthoquinones, menathioquinones (2-methylthio-1,4-naphthoquinone) are present [31-35]. The polar lipids reported in members of the genera *Aquifex, Hydrogenobaculum*, *Hydrogenothermus* and *Thermocrinis* are also characteristic, with an unusual aminopentanetetrol phospholipid derivative being present in all strains examined [36,37]. Where detailed analyses have been carried out phosphatidylinositol has also been reported [37]. Stöhr *et al.* labeled these lipids PNL and PL1 respectively [31].

## Genome sequencing and annotation

### Genome project history

This organism was selected for sequencing on the basis of its phylogenetic position, and is part of the *** G****enomic* *** E****ncyclopedia of* *** B****acteria and* *** A****rchaea * project [38]. The genome project is deposited in the Genome OnLine Database [18] and the complete genome sequence is deposited in GenBank. Sequencing, finishing and annotation were performed by the DOE Joint Genome Institute (JGI). A summary of the project information is shown in Table 2.

**Table 2:** Genome sequencing project information

**MIGS ID**	**Property**	**Term**
MIGS-31	Finishing quality	Finished
MIGS-28	Libraries used	Two 454 pyrosequence libraries, standard and pairs end (17 kb insert size)
MIGS-29	Sequencing platforms	454 Titanium, Illumina GAii
MIGS-31.2	Sequencing coverage	52.9× 454 Titanim; 298× Illumina
MIGS-30	Assemblers	Newbler, Velvet, phrap
MIGS-32	Gene calling method	Prodigal, GenePRIMP
	INSDC ID	CP001931
	Genbank Date of Release	February 19, 2010
	GOLD ID	Gc01206
	NCBI project ID	37275
	Database: IMG-GEBA	2502082116
MIGS-13	Source material identifier	DSM 14484
	Project relevance	Tree of Life, GEBA

### Growth conditions and DNA isolation

*T. albus* HI 11/12^T^, DSM 14484, was grown in DSMZ medium 887 (OS Medium) [39] at 80°C. DNA was isolated from 1-1.5 g of cell paste using MasterPure Gram-positive Kit (Epicentre MGP04100) with a modified protocol for cell lysis, using an additional 5 µl mutanolysin to standard lysis solution, and one hour incubation on ice after the MPC-step.

### Genome sequencing and assembly

The genome of strain HI 11/12^T^ was sequenced using a combination of Illumina [40] and 454. An Illumina GAii shotgun library with reads of 447 Mb, a 454 Titanium draft library with average read length of 287 bases, and a paired end 454 library with average insert size of 17 Kb were generated for this genome. All general aspects of library construction and sequencing can be found at http://www.jgi.doe.gov/. Illumina sequencing data were assembled with VELVET [41], and the consensus sequences were shredded into 1.5 kb overlapped fake reads and assembled together with the 454 data. Draft assemblies were based on 79 Mb 454 draft data. Newbler parameters were -consed -a 50 -l 350 -g -m -ml 20. The initial assembly contained six contigs in one scaffold. We converted the initial 454 assembly into a phrap assembly by making *fake* reads from the consensus, collecting the read pairs in the 454 paired end library. The Phred/Phrap/Consed software package (www.phrap.com) was used for sequence assembly and quality assessment in the subsequent finishing process. After the shotgun stage, reads were assembled with parallel phrap. Possible mis-assemblies were corrected with gapResolution (unpublished; http://www.jgi.doe.gov/), Dupfinisher or sequencing cloned bridging PCR fragments with subcloning or transposon bombing [42]. Gaps between contigs were closed by editing in Consed, by PCR and by Bubble PCR primer walks (J-F. Chan, unpublished). A total of 68 additional reactions were necessary to close gaps and to raise the quality of the finished sequence. The completed genome sequence had an error rate of less than 1 in 100,000 bp

### Genome annotation

Genes were identified using Prodigal [43] as part of the Oak Ridge National Laboratory genome an-notation pipeline, followed by a round of manual curation using the JGI GenePRIMP pipeline [44]. The predicted CDSs were translated and used to search the National Center for Biotechnology Information (NCBI) nonredundant database, UniProt, TIGRFam, Pfam, PRIAM, KEGG, COG, and InterPro databases. Additional gene prediction analysis and manual functional annotation was performed within the Integrated Microbial Genomes Expert Review (IMG-ER) platform [45].

## Genome properties

**Figure 3:**
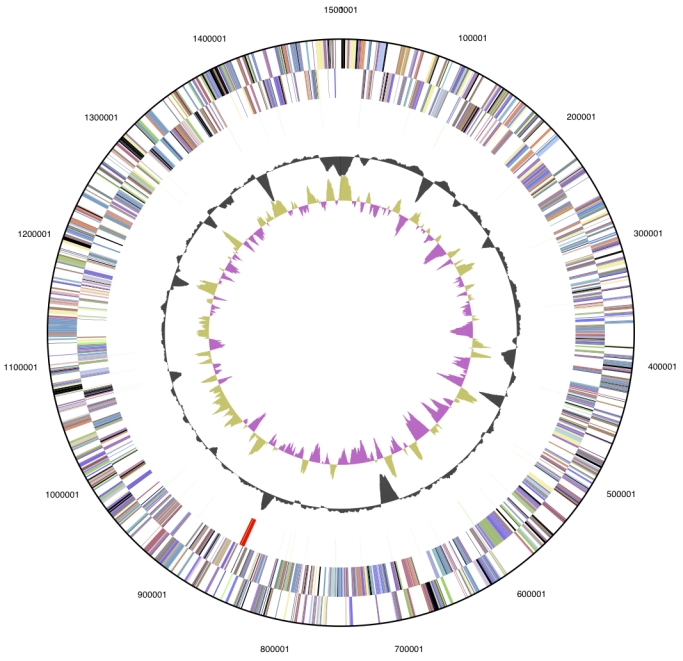
Graphical circular map of the genome. From outside to the center: Genes on forward strand (color by COG categories), Genes on reverse strand (color by COG categories), RNA genes (tRNAs green, rRNAs red, other RNAs black), GC content, GC skew.

The genome consists of a 1,500,577 bp long chromosome with a 46.9% GC content (Table 3 and Figure 3). Of the 1,650 genes predicted, 1,593 were protein coding genes, and 47 RNAs; 10 pseudogenes were identified. The majority of the protein-coding genes (75.2%) were assigned with a putative function while those remaining were annotated as hypothetical proteins. The distribution of genes into COGs functional categories is presented in Table 4.

**Table 3:** Genome Statistics

**Attribute**	**Value**	**% of Total**
Genome size (bp)	1,500,577	100.00%
DNA coding region (bp)	1,459,457	97.26%
DNA G+C content (bp)	704,229	46.93%
Number of replicons	1	
Extrachromosomal elements	0	
Total genes	1,650	100.00%
RNA genes	47	2.85%
rRNA operons	1	
Protein-coding genes	1,603	97.15%
Pseudo genes	10	0.61%
Genes with function prediction	1,241	75.21%
Genes in paralog clusters	124	7.52%
Genes assigned to COGs	1,316	79.76%
Genes assigned Pfam domains	1,333	80.79%
Genes with signal peptides	243	14.73%
Genes with transmembrane helices	322	19.52%
CRISPR repeats	4	

**Table 4:** Number of genes associated with the general COG functional categories

**Code**	**value**	**%age**	**Description**
J	131	8.2	Translation, ribosomal structure and biogenesis
A	0	0.0	RNA processing and modification
K	45	2.8	Transcription
L	80	5.0	Replication, recombination and repair
B	2	0.1	Chromatin structure and dynamics
D	0	0.0	Cell cycle control, mitosis and meiosis
Y	0	0.0	Nuclear structure
V	11	0.7	Defense mechanisms
T	47	2.9	Signal transduction mechanisms
M	111	6.9	Cell wall/membrane biogenesis
N	57	3.6	Cell motility
Z	0	0.0	Cytoskeleton
W	0	0.0	Extracellular structures
U	58	3.6	Intracellular trafficking and secretion
O	78	4.9	Posttranslational modification, protein turnover, chaperones
C	140	8.7	Energy production and conversion
G	48	3.0	Carbohydrate transport and metabolism
E	118	7.4	Amino acid transport and metabolism
F	49	3.1	Nucleotide transport and metabolism
H	96	6.0	Coenzyme transport and metabolism
I	39	2.4	Lipid transport and metabolism
P	74	4.6	Inorganic ion transport and metabolism
Q	16	1.0	Secondary metabolites biosynthesis, transport and catabolism
R	148	9.2	General function prediction only
S	79	4.9	Function unknown
-	334	20.8	Not in COGs

## Insights from the genome sequence

With only very few papers published on the organism [1,2], and only one gene sequence (16S rRNA) available in GenBank from strain HI 11/12^T^, a comparison of already known sequences to the here presented novel genomic data is rather meager for *T. albus*. As shown in Figure 1, there are presently no other type strain genomes from the *Aquificaceae* available either to allow a meaningful comparative genomics analysis. This might change when other type/neotype strains of species within the genus *Thermocrinis* which are also part of the ***G****enomic* ***E****ncyclopedia of* ***B****acteria and* ***A****rchaea* project [38] will become available in the near future.

## References

[1] Eder W Huber R New isolates and physiological properties of the *Aquificales* and description of *Thermocrinis albus* sp. nov. Extremophiles 2002; 6:309-318 10.1007/s00792-001-0259-y 12215816

[2] Huber R Eder W Heldwein S Wanner G Huber H Rachel R Stetter KO *Thermocrinis ruber* gen. nov., sp. nov., a pink-filament-forming hyperthermophilic bacterium isolated from Yellowstone National Park. Appl Environ Microbiol 1998; 64:3576-3583 9758770 10.1128/aem.64.10.3576-3583.1998 PMC106467

[3] Euzéby JP List of bacterial names with standing in nomenclature: A folder available on the Internet. Int J Syst Bacteriol 1997; 47:590-592 10.1099/00207713-47-2-590 9103655

[4] Hall JR Mitchell KR Jackson-Weaver O Kooser AS Cron BR Crossey LJ Takacs-Vesbach CD Molecular characterization of the diversity and distribution of a thermal spring microbial community by using rRNA and metabolic genes. Appl Environ Microbiol 2008; 74:4910-4922 10.1128/AEM.00233-08 18539788 PMC2519350

[5] Connon SA Koski AK Neal AL Wood SA Magnuson TS Ecophysiology and geochemistry of microbial arsenic oxidation within a high arsenic, circumneutral hot spring system of the Alvord Desert. FEMS Microbiol Ecol 2008; 64:117-128 10.1111/j.1574-6941.2008.00456.x 18318711

[6] Hamamura N Macur RE Korf S Ackerman G Taylor WP Kozubal M Reysenbach AL Inskeep WP Linking microbial oxidation of arsenic with detection and phylogenetic analysis of arsenite oxidase genes in diverse geothermal environments. Environ Microbiol 2009; 11:421-431 10.1111/j.1462-2920.2008.01781.x 19196273

[7] Hügler M Huber H Molyneaux SJ Vetriani C Sievert SM Autotrophic CO_2_ fixation via the reductive tricarboxylic acid cycle in different lineages within the phylum *Aquificae*: evidence for two ways of citrate cleavage. Environ Microbiol 2007; 9:81-92 10.1111/j.1462-2920.2006.01118.x 17227414

[8] Deckert G Warren PV Gaasterland T Young WG Lennox AL Graham DE Overbeek R Snead MA Keller M Aujay M The complete genome of the hyperthermophilic bacterium *Aquifex aeolicus.* Nature 1998; 392:353-358 10.1038/32831 9537320

[9] Klenk HP The genomics of a hot-water maker. Nat Genet 1998; 19:4-6 10.1038/ng0598-4 9590275

[10] Huber R, Stetter KO. Genus I. *Aquifex* Huber and Stetter 1992e, 656^VP^ (Effective publication: Huber and Stetter *in* Huber, Wilharm, Huber, Trincone, Burggraf, König, Rachel, Rockinger, Fricke and Stetter 1992b, 349). *In*: Boone DR, Castenholz WR, Garrity GM (eds), *Bergey's Manual of Systematic Bacteriology*, second edition, vol. 1 (The *Archaea* and the deeply branching and phototrophic *Bacteria*), Springer, New York, 2001, pp. 360-362.

[11] Skirnisdottir S Hreggvidsson GO Hjorleifsdottir S Marteinsson VT Petursdottir SK Holst O Kristjansson JK Influence of sulfide and temperature on species composition and community structure of hot spring microbial mats. Appl Environ Microbiol 2000; 66:2835-2841 10.1128/AEM.66.7.2835-2841.2000 10877776 PMC92081

[12] Marteinsson VT Hauksdottir S Hobel CF Kristmannsdottir H Hreggvidsson GO Kristjansson JK Phylogenetic diversity analysis of subterranean hot springs in Iceland. Appl Environ Microbiol 2001; 67:4242-4248 10.1128/AEM.67.9.4242-4248.2001 11526029 PMC93153

[13] Chun J Lee JH Jung Y Kim M Kim S Kim BK Lim YW EzTaxon: a web-based tool for the identification of prokaryotes based on 16S ribosomal RNA gene sequences. Int J Syst Evol Microbiol 2007; 57:2259-2261 10.1099/ijs.0.64915-0 17911292

[14] Castresana J Selection of conserved blocks from multiple alignments for their use in phylogenetic analysis. Mol Biol Evol 2000; 17:540-552 10742046 10.1093/oxfordjournals.molbev.a026334

[15] Lee C Grasso C Sharlow MF Multiple sequence alignment using partial order graphs. Bioinformatics 2002; 18:452-464 10.1093/bioinformatics/18.3.452 11934745

[16] Stamatakis A Hoover P Rougemont J A rapid bootstrap algorithm for the RAxML web servers. Syst Biol 2008; 57:758-771 10.1080/10635150802429642 18853362

[17] Pattengale ND Alipour M Bininda-Emonds ORP Moret BME Stamatakis A How many bootstrap replicates are necessary? Lect Notes Comput Sci 2009; 5541:184-200 10.1007/978-3-642-02008-7_13 20377449

[18] Liolios K Chen IM Mavromatis K Tavernarakis N Hugenholtz P Markowitz VM Kyrpides NC The Genomes On Line Database (GOLD) in 2009: status of genomic and metagenomic projects and their associated metadata. Nucleic Acids Res 2010; 38:D346-D354 10.1093/nar/gkp848 19914934 PMC2808860

[19] Field D Garrity G Gray T Morrison N Selengut J Sterk P Tatusova T Thomson N Allen MJ Angiuoli SV The minimum information about a genome sequence (MIGS) specification. Nat Biotechnol 2008; 26:541-547 10.1038/nbt1360 18464787 PMC2409278

[20] Woese CR Kandler O Wheelis ML Towards a natural system of organisms: proposal for the do-mains *Archaea, Bacteria*, and *Eucarya.* Proc Natl Acad Sci USA 1990; 87:4576-4579 10.1073/pnas.87.12.4576 2112744 PMC54159

[21] Reysenbach AL. Class I, *Aquificae* phyl. nov. In: Garrity GM, Boone DR, Castenholz RW (eds), *Bergey’s Manual of Systematic Bacteriology*, Second Edition, Vol. 1, Springer, NY, 2001, p. 359.

[22] Reysenbach AL. Order I, Aquificae class nov. In: Garrity GM, Boone DR, Castenholz RW (eds), *Bergey’s Manual of Systematic Bacteriology*, Second Edition, Vol. 1, Springer, NY, 2001, p. 359.

[23] Reysenbach AL. Family I, *Aquificaceae* fam. nov. In: Garrity GM, Boone DR, Castenholz RW (eds), *Bergey’s Manual of Systematic Bacteriology*, Second Edition, Vol. 1, Springer, NY, 2001, p. 360.

[24] Caldwell SL Liu Y Ferrera I Beveridge T Reysenbach A-L *Thermocrinis minervae* sp. nov., a hydrogen- and sulfur-oxidizing, thermophilic member of the *Aquificales* from a Costa Rican terrestrial hot spring. Int J Syst Evol Microbiol (In press). 19651724 10.1099/ijs.0.010496-0

[25] Classification of *Bacteria* and *Archaea* in risk groups Technical rules for biological agents www.baua.de TRBA 466.

[26] Ashburner M Ball CA Blake JA Botstein D Butler H Cherry JM Davis AP Dolinski K Dwight SS Eppig JT Gene Ontology: tool for the unification of biology. Nat Genet 2000; 25:25-29 10.1038/75556 10802651 PMC3037419

[27] Plötz BM Lindner B Stetter KO Holst OJ Characterization of a novel lipid A containing D-galacturonic acid that replaces phosphate residues. The structure of the lipid A of the lipopolysaccharide from the hyperthermophilic bacterium *Aquifex pyrophilus.* Biol Chem 2000; 275:11222-11228 10.1074/jbc.275.15.11222 10753930

[28] Mamat U Schmidt HE Lindner B Fukase K Hanuszkiewicz A Wu J Meredith TC Woodard RW Hilgenfeld R Mesters JR WaaA of the hyperthermophilic bacterium *Aquifex aeolicus* is a monofunctional 3-deoxy-d-manno-oct-2-ulosonic acid transferase involved in lipopolysaccharide biosynthesis. J Biol Chem 2009; 284:22248-22262 10.1074/jbc.M109.033308 19546212 PMC2755949

[29] Hosoya R Hamana K Niitsu M Itoh T Polyamine analysis for chemotaxonomy of thermophilic eubacteria: Polyamine distribution profiles within the orders *Aquificales, Thermotogales, Thermodesulfobacteriales, Thermales, Thermoanaerobacteriales, Clostridiales* and *Bacillales.* J Gen Appl Microbiol 2004; 50:271-287 10.2323/jgam.50.271 15747232

[30] Jahnke LL Eder W Huber R Hope JM Hinrichs KU Hayes JM Des Marais DJ Cady SL Summons RE Signature lipids and stable carbon isotope analyses of Octopus Spring hyperthermophilic communities compared with those of aquificales representatives. Appl Environ Microbiol 2001; 67:5179-5189 10.1128/AEM.67.11.5179-5189.2001 11679343 PMC93288

[31] Stöhr R Waberski A Völker H Tindall BJ Thomm M *Hydrogenothermus marinus* gen. nov., sp. nov., a novel thermophilic hydrogen-oxidizing bacterium, recognition of *Calderobacterium hydrogenophilum* as a member of the genus *Hydrogenobacter a*nd proposal of the reclassification of *Hydrogenobacter acidophilus* as *Hydrogenobaculum acidophilum* gen. nov., comb. nov., in the phylum *‘Hydrogenobacter/Aquifex’.* Int J Syst Evol Microbiol 2001; 51:1853-1862 11594618 10.1099/00207713-51-5-1853

[32] Ishii M Kawasumi T Igarashi Y Kodama T Minoda Y 2-Methylthio-1,4-naphthoquinone, a new quinone from an extremely thermophilic hydrogen bacterium. Agric Biol Chem 1983; 47:167-169 10.1128/jb.169.6.2380-2384.1987 PMC212068 3584059

[33] Ishii M Kawasumi T Igarashi Y Kodama T Minoda Y 2-Methylthio-1,4-naphthoquinone, a unique sulfur containing quinone from a thermophilic hydrogen-oxidizing bacterium, *Hydrogenobacter thermophilus.* J Bacteriol 1987; 169:2380-2384 3584059 10.1128/jb.169.6.2380-2384.1987 PMC212068

[34] Nishihara H Igarashi Y Kodama T A new isolate of *Hydrogenobacter*, an obligately chemolithoautotrophic, thermophilic, halophilic and aerobic hydrogen-oxidizing bacterium from seaside saline hot spring. Arch Microbiol 1990; 153:294-298 10.1007/BF00249085

[35] Shima S Suzuki KI *Hydrogenobacter acidophilus* *sp. nov.,* a thermoacidophilic, aerobic, hydrogen-oxidizing bacterium requiring elemental sulfur for growth. Int J Syst Bacteriol 1993; 43:703-708 10.1099/00207713-43-4-703

[36] Sturt HF Summons RE Smith K Elvert M Hinrichs K-U. Intact polar membrane lipids in prokaryotes and sediments deciphered by high-performance liquid chromatography/electrospray ionization multistage mass spectrometry — new biomarkers for biogeochemistry and microbial ecology. Rapid Commun Mass Spectrom 2004; 18:617-628 10.1002/rcm.1378 15052572

[37] Yoshino J Sugiyama Y Sakuda S Kodama T Nagasawa H Ishii M Igarashi Y Chemical structure of a novel aminophospholipid from *Hydrogenobacter thermophilus* strain TK-6. J Bacteriol 2001; 183:6302-6304 10.1128/JB.183.21.6302-6304.2001 11591674 PMC100120

[38] Wu D Hugenholtz P Mavromatis K Pukall R Dalin E Ivanova NN Kunin V Goodwin L Wu M Tindall BJ A phylogeny-driven genomic encyclopaedia of *Bacteria* and *Archaea*. Nature 2009; 462:1056-1060 10.1038/nature08656 20033048 PMC3073058

[39] List of growth media used at DSMZ: http://www.dsmz.de/microorganisms/ me-dia_list.php

[40] Bennett S Solexa Ltd. Pharmacogenomics 2004; 5:433-438 10.1517/14622416.5.4.433 15165179

[41] Zerbino DR Birney E Velvet: algorithms for de novo short read assembly using de Bruijn graphs. Genome Res 2008; 18:821-829 10.1101/gr.074492.107 18349386 PMC2336801

[42] Sims D Brettin T Detter J Han C Lapidus A Copeland A Glavina Del Rio T Nolan M Chen F Lucas S Complete genome sequence of *Kytococcus sedentarius* type strain (541^T^). Stand Genomic Sci 2009; 1:12-20 10.4056/sigs.761 PMC3035214 21304632

[43] Hyatt D Chen GL Locascio PF Land ML Larimer FW Hauser LJ Prodigal Prokaryotic Dynamic Programming Genefinding Algorithm. BMC Bioinformatics 2010; 11**:** 119 10.1186/1471-2105-11-119 20211023 PMC2848648

[44] Pati A Ivanova N Mikhailova N Ovchinikova G Hooper SD Lykidis A Kyrpides NC GenePRIMP: A Gene Prediction Improvement Pipeline for microbial genomes. Nat Methods (In press). 10.1038/nmeth.1457 20436475

[45] Markowitz VM Ivanova NN Chen IMA Chu K Kyrpides NC IMG ER: a system for microbial genome annotation expert review and curation. Bioinformatics 2009; 25:2271-2278 10.1093/bioinformatics/btp393 19561336

